# Wasp Venom Ameliorates Scopolamine-Induced Learning and Memory Impairment in Mice

**DOI:** 10.3390/toxins14040256

**Published:** 2022-04-04

**Authors:** Ji Hyeong Chae, Jisun Oh, Ji Sun Lim, Yoon Ah Jeong, Hyun Seok Yun, Chan Ho Jang, Hyo Jung Kim, Jong-Sang Kim

**Affiliations:** 1 Department of Integrative Biology, Kyungpook National University, Daegu 41566, Korea; chski10004@knu.ac.kr; 2 New Drug Development Center, Daegu-Gyeongbuk Medical Innovation Foundation, Daegu 41061, Korea; joh@kmedihub.re.kr; 3 Department of Nuclear Medicine, Keimyung University Dongsan Hospital, Daegu 42601, Korea; lzsunny@daum.net; 4 School of Food Science and Biotechnology, Kyungpook National University, Daegu 41566, Korea; bory1506@daum.net (Y.A.J.); solideo0116@naver.com (H.S.Y.); cksghwkd7@gmail.com (C.H.J.); 5 Department of Korean Medicine Development, National Institute for Korean Medicine Development, Gyeongsan 38540, Korea; indersee31@nikom.or.kr

**Keywords:** *Vespa velutina*, wasp venom, bee venom, memory impairment, nuclear factor erythroid 2-related factor 2 (Nrf2), antioxidant enzymes, Alzheimer’s disease

## Abstract

This study investigated the effects of wasp venom (WV) from the yellow-legged hornet, *Vespa velutina*, on scopolamine (SCO)-induced memory deficits in mice, as well as the antioxidant activity in HT22 murine hippocampal neuronal cells in parallel comparison with bee venom (BV). The WV was collected from the venom sac, freeze-dried. Both venoms exhibited free radical scavenging capabilities in a concentration-dependent manner. In addition, the venom treatment enhanced cell viability at the concentrations of ≤40 µg/mL of WV and ≤4 µg/mL of BV in glutamate-treated HT22 cells, and increased the transcriptional activity of the antioxidant response element (ARE), a *cis*-acting enhancer which regulates the expression of nuclear factor erythroid 2-related factor 2 (Nrf2)-downstream antioxidant enzymes. Concurrently, WV at 20 µg/mL significantly increased the expression of a key antioxidant enzyme heme oxygenase 1 (HO-1) in HT22 cells despite no significant changes observed in the nuclear level of Nrf2. Furthermore, the intraperitoneal administration of WV to SCO-treated mice at doses ranged from 250 to 500 µg/kg body weight ameliorated memory impairment behavior, reduced histological injury in the hippocampal region, and reduced oxidative stress biomarkers in the brain and blood of SCO-treated mice. Our findings demonstrate that WV possess the potential to improve learning and memory deficit in vivo while further study is needed for the proper dose and safety measures and clinical effectiveness.

## 1. Introduction

Wasp venom (WV) has been reported to contain diverse compounds, consisting of amines, small peptides (mastoparan, eumenitin, eumenitin-R, rumenitin-F, EpVP, decoralin, anoplin, etc.), various enzymes (hyaluronidase, α-glucosidase, phosphatase, phospholipase A2, phospholipase B, etc.), allergens and toxins [1]. Certain compounds present in WV have been suggested to have biologically beneficial effects exhibiting antimicrobial [2,3], anticancer [4], and antiinflammatory activities [5].

In particular, our previous study has demonstrated that WV which was isolated from *Vespa velutina nigrithorax* (commonly called the invasive yellow-legged hornet) inhibited the microglial activation through suppressing the nuclear factor kappa B (NFκB) pathway in vitro [6]. This may have implications for therapeutic intervention using WV in neurodegenerative disorders since the chronic activation of microglial cells contributes to the pathophysiology of Alzheimer’s disease (AD), Parkinson’s disease, and amyotrophic lateral sclerosis [6,7].

Considering that NFκB and nuclear factor erythroid 2-related factor 2 (Nrf2) signaling pathways generally operate in an opposing manner [6], we herein hypothesized that WV could promote the Nrf2 signaling pathway. This hypothesis was supported by our preliminary results indicating that WV may have direct antioxidative activity by scavenging free radicals and inducing the expression of antioxidant enzymes, which is one of the promising strategies to counteract neurodegenerative progression.

Multiple studies demonstrated that oxidative stress primarily contributes to neuronal death in neurodegenerative diseases [8,9,10]. Reactive oxygen species (ROS) production can be induced by microglial activation in the brain [11]. Especially, increased oxidative stress can upregulate expression of the amyloid-β precursor protein (APP) and increase the β- and γ-secretase activities in AD brain [12], which subsequently mediates cleavage of APP and causes accumulation of an extracellular oligomeric peptide, amyloid-β (Aβ). The Aβ deposition affects ion channel properties in the plasma membrane and enzyme activities of intracellular kinase, such as GSK3β and Cdk5, which cause hyperphosphorylation of tau proteins and the subsequent synaptic dysfunction and neuronal loss [8]. Thus, oxidative stress is an essential part of the pathological process of AD and is closely associated with amyloid pathology by formation of serious pathophysiological cycles [8]. Oxidative stress is not only an essential pathological marker of AD, but also serves as a potential treatment target.

The elevated level of oxidative stress is involved in neurodegeneration and cognitive dysfunction in experimental animals as well as human [13]. Scopolamine (SCO), a non-selective antagonist of muscarinic acetylcholine receptor, is known to provoke oxidative stress in the brain and induce learning and memory impairment [14,15]; therefore, it is often used experimentally to produce an amnesia or a dementia rodent model.

Hence, activation of the Nrf2 signaling pathway is expected to improve cognitive function by suppressing abnormal ROS generated in brain tissue, particularly the hippocampus. This study was conducted to examine whether WV could improve or restore a SCO-induced cognitive impairment in mice by upregulating the Nrf2-dependent antioxidant enzyme.

## 2. Results

### 2.1. In Vitro Antioxidant Activity of WV

**Figure 1:**
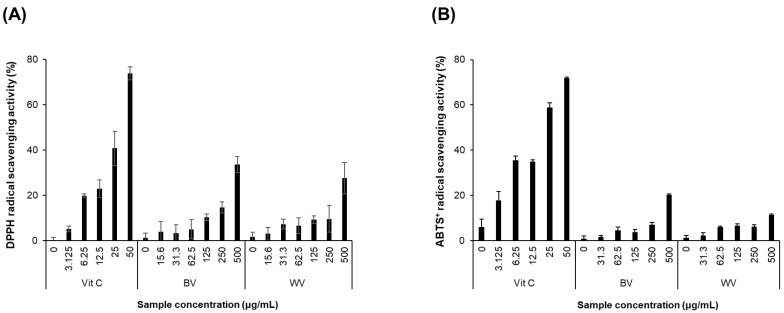
**DPPH and ABTS^+^ radical scavenging activities of WV.** (**A**) DPPH and (**B**) ABTS^+^ radical scavenging activities were determined at various concentrations (15.6–500 μg/mL) of WV in parallel comparison with BV. Results are expressed as mean ± SD (*n* = 3). Values not sharing a common alphabetic character represent a statistically significant difference among experimental groups (*p* < 0.05). Vit C, ascorbic acid (as a positive control); BV, bee venom; WV, wasp venom.

WV showed concentration-dependent DPPH and ABTS^+^ radical scavenging capability which was similar to BV (Figure 1). The scavenging activity of WV was observed more prominent for DPPH than ABTS^+^ radical.

### 2.2. WV Protected against Glutamate-Induced Cytotoxicity

**Figure 2:**
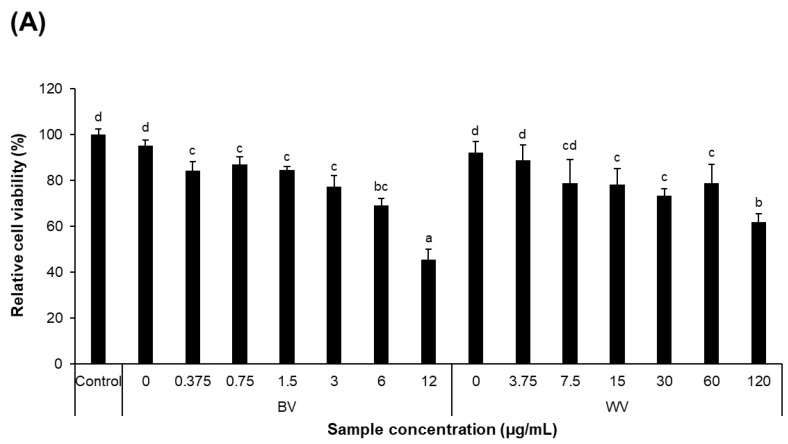

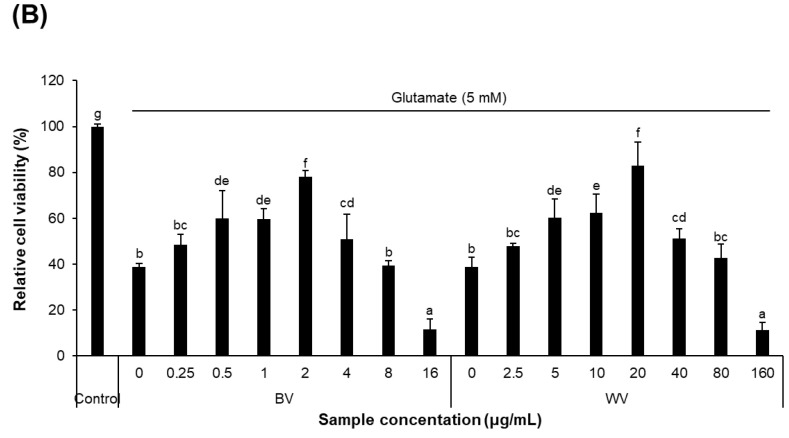
**Cytotoxicity of BV and WV in HT22 cells.** Murine hippocampal neuronal cell line, HT22, was treated with various concentrations of BV (0.375–12 μg/mL) and WV (3.75–120 μg/mL) in the absence (**A**) and presence of 5 mM glutamate (**B**) for 24 h. The cytotoxicity was assayed using the CCK-8. Values and error bars are presented as mean ± SEM (*n* = 3). Statistical analysis was performed by one-way ANOVA, followed by Duncan’s multiple range test. Values not sharing a common alphabetic character represent a statistically significant difference among experimental groups (*p* < 0.05). BV, bee venom; WV, wasp venom.

Cytotoxicity assay showed that IC_50_ values of WV and BV were > 120 μg/mL and 6–12 μg/mL, respectively, indicating that WV was significantly less toxic than BV in HT22 mouse hippocampal neuronal cells (Figure 2A). When treated with glutamate, causing cytotoxicity in HT22 cells by raising intracellular oxidative stress [16,17,18,19], WV at the concentrations of ≤40 µg/mL and BV at ≤4 µg/mL inhibited glutamate-induced cellular toxicity (Figure 2B). However, WV at 80 μg/mL or higher was not effective in alleviating glutamate-induced cytotoxicity, and rather found to be toxic.

### 2.3. WV Increased Antioxidant Response Element (ARE)-Luciferase Activity

**Figure 3:**
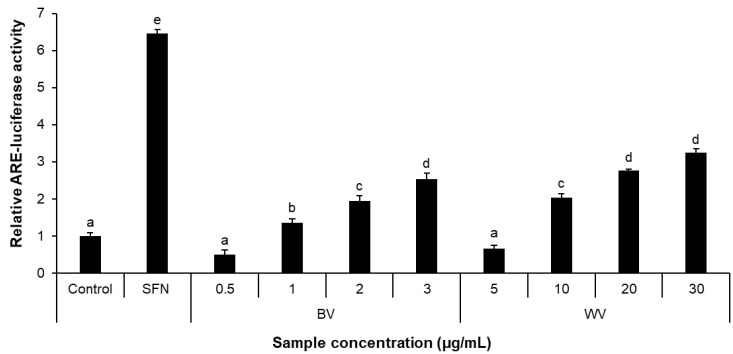
**ARE-luciferase induction activity of WV and BV in HT22-ARE cells**. HT22-ARE cells were treated with the designated concentrations of WV and BV. SFN (1 μM), an ARE activator, was used as a positive control. Results are expressed as mean ± SEM (*n* = 3). Statistical analysis was performed by one-way ANOVA, followed by Duncan’s multiple range test. Values not sharing a common alphabetic character represent a statistically significant difference among experimental groups (*p* < 0.05). SFN, sulforaphane; BV, bee venom; WV, wasp venom.

The transcriptional activity of ARE, a *cis*-acting enhancer which regulates the expression of Nrf2-downstream antioxidant enzymes, was examined using HT22-ARE cells harboring the luciferase reporter-encoding gene following the ARE sequence in the transduced vector. WV and BV at the concentrations nontoxic to HT22 cells were found to increase luciferase reporter activity in a concentration-dependent manner (Figure 3), indicating its potential to promote nuclear translocation of Nrf2 and induce the transcription of a set of antioxidant enzyme genes.

### 2.4. WV Upregulated Cytoplasmic HO-1 Level Downstream of Nrf2

**Figure 4:**
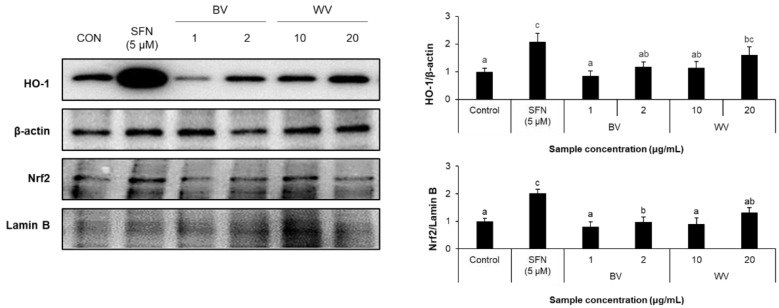
**Effect of WV on the expression of antioxidant proteins in HT22 cells.** The expression levels of Nrf2 and HO-1 in HT22 cells were measured by western blot analysis and normalized by lamin B and β-actin, respectively. SFN (5 μM) was used as a positive control. Results are expressed as mean ± SEM (*n* = 3). Statistical analysis was performed by one-way ANOVA, followed by Duncan’s multiple range test. Values not sharing a common alphabetic character represent a statistically significant difference among experimental groups (*p* < 0.05). SFN, sulforaphane; BV, bee venom; WV, wasp venom.

Consistently, WV treatment at 20 μg/mL resulted in a significant induction of HO-1, a type of inducible antioxidant enzyme, although the nuclear level of Nrf2 was marginally affected (Figure 4).

### 2.5. WV Decreased Cellular ROS Level

**Figure 5:**
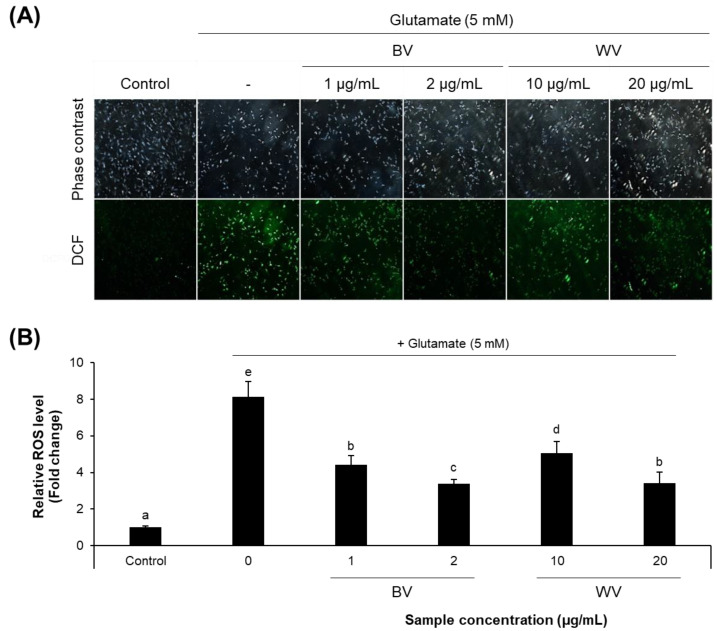
**Reduction of intracellular ROS level by BV and WV in HT22 cells.** (**A**) Intracellular ROS levels were visualized under fluorescence microscopy. (**B**) The fluorescence intensity was measured using a fluorescence microplate reader and relatively quantified to the control. The results are expressed as means ± SEM (*n* = 3). Statistical analysis was performed by one-way ANOVA, followed by Duncan’s multiple range test. Values not sharing a common alphabetic character indicate a statistically significant difference among experimental groups (*p* < 0.05). BV, bee venom; WV, wasp venom.

The DCF assay showed that treatment of HT22 cells with glutamate, a cytotoxic agent, increased intracellular ROS level as expected (Figure 5). However, the WV or BV treatment significantly lowered the ROS level in glutamate-treated cells in a concentration-dependent manner.

### 2.6. WV Improved Learning and Memory in SCO-Treated Mouse Model

**Figure 6:**
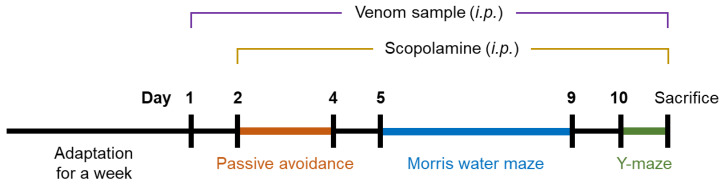
Experimental schedule for behavioral study using C57BL/6J mice.

A total of 56 C57BL/6J mice were randomly allocated into 8 different groups (Table 1). WV or BV were intraperitoneally administered in combination with SCO treatment every day for 10 days (Figure 6).

**Figure 7:**
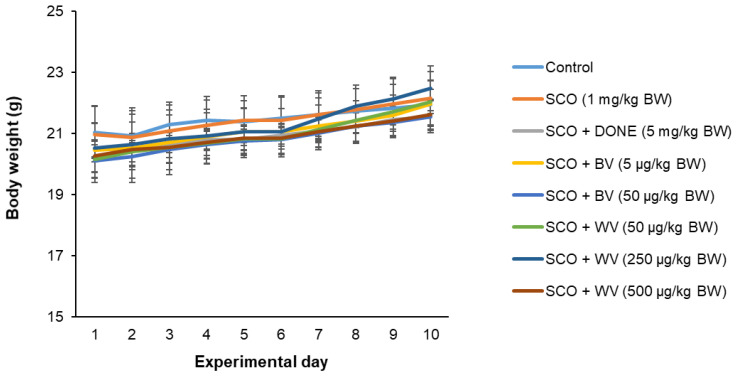
**Effect of intraperitoneal injection of WV on mouse body weight.** After 1-week adaptation, mice were intraperitoneally injected with SCO at a dose of 1 mg/kg BW every single day for a total of 10 days and were intraperitoneally treated with BV (5 or 50 μg/kg BW) or WV (50, 250, or 500 μg/kg BW) 15 h prior to SCO treatment. The average BW was monitored during the entire experimental period. Results are expressed as means ± SD (*n* = 7). Statistical analysis was performed by one-way ANOVA, followed by Duncan’s multiple range test. SCO, scopolamine; DONE, donepezil; BV, bee venom; WV, wasp venom.

As a result of regular monitoring, administration of BV or WV and treatment with SCO did not have a significant effect on the BW of mice during the experimental period (Figure 7).

**Figure 8:**
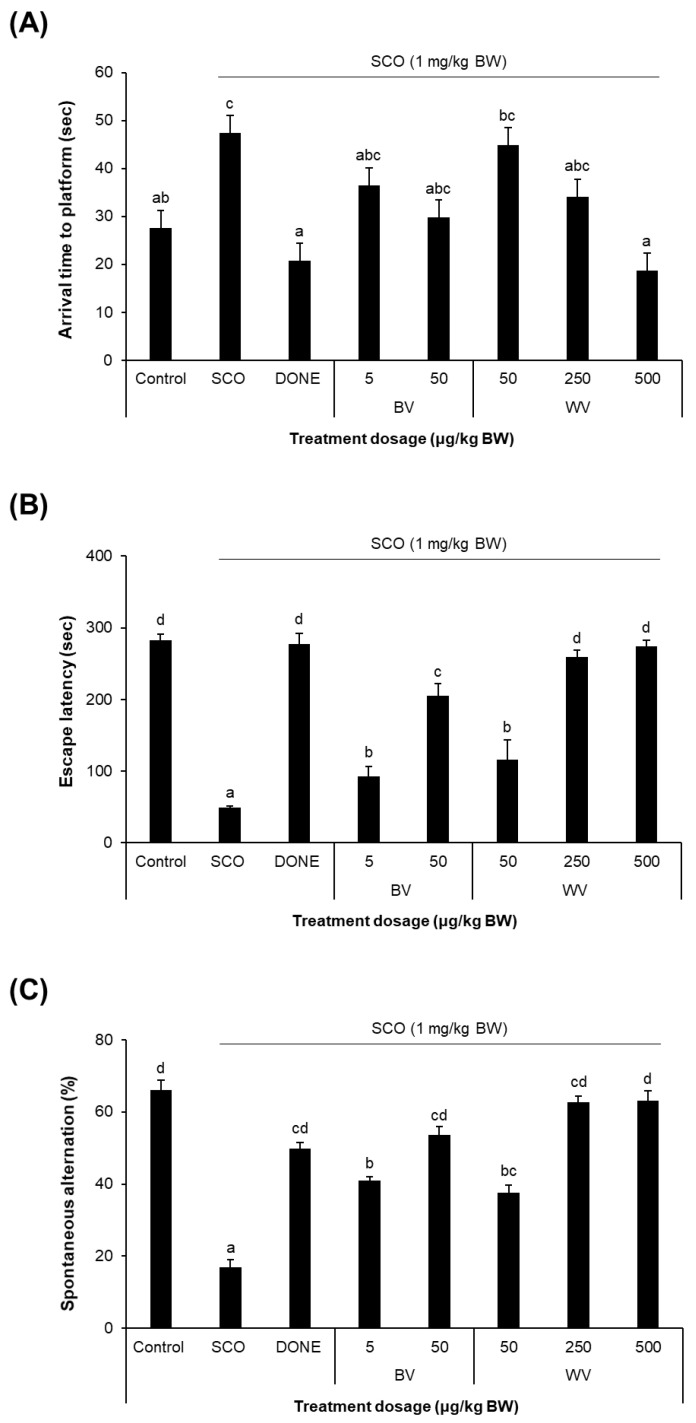
**Effect of BV and WV against SCO-induced learning and memory impairment behavior.** (**A**) Morris water maze task; (**B**) Passive avoidance task; (**C**) Y-maze task. Results are expressed as means ± SD (*n* = 7). Statistical analysis was performed by one-way ANOVA, followed by Duncan’s multiple range test. Values not sharing a common alphabetic character represent a significant difference among experimental groups (*p* < 0.05). SCO, scopolamine; DONE, donepezil; BV, bee venom; WV, wasp venom.

The Morris water maze task demonstrated that the treatment with WV (500 μg/kg bw) or donepezil (5 mg/kg BW) improved SCO-induced spatial learning and memory impairment in both tested concentrations (Figure 8A). The passive avoidance task showed that WV (50 μg/kg BW) or BV (5 μg/kg BW) ameliorated the SCO-induced associative learning and memory deficit (Figure 8B). Moreover, administration of WV at 250 μg/kg BW or higher displayed similar memory improvement to donepezil, a positive control. The WV administration also improved short-term spatial memory as tested the willingness of mice to explore new environments in the Y-maze, which was diminished by the SCO treatment (Figure 8C). The parts of the brain involved in such learning and memory include the hippocampus, basal forebrain, and prefrontal cortex [20].

### 2.7. WV Protected Hippocampal Region from SCO-Induced Damage

**Figure 9:**
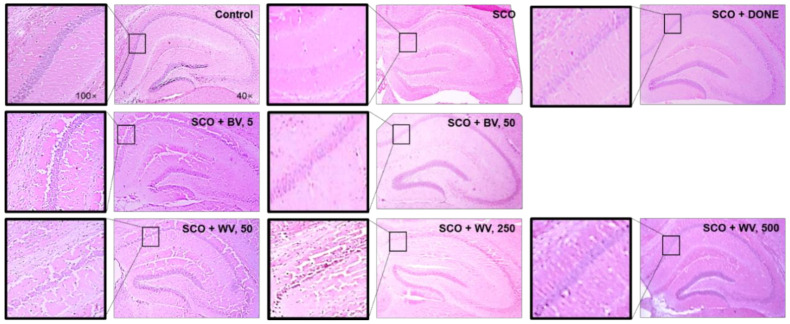
**Effect of BV and WV against SCO-induced neuronal damage in hippocampal CA1 region of mice**. Control, no treatment; SCO, treatment with SCO alone (1 mg/kg BW); SCO + DONE, treatment with SCO and DONE; SCO + BV, 5, treatment with SCO and BV at 5 μg/kg BW; SCO + BV, 50, treatment with SCO and BV at 50 μg/kg BW; SCO + WV, 50, treatment with SCO and WV at 50 μg/kg BW; SCO + WV, 250, treatment with SCO and WV at 250 μg/kg BW; SCO + WV, 500, treatment with SCO and WV at 500 μg/kg BW. Representative pictures of each experimental group were presented. Pictures in the left panel were magnified by 100-fold while those in the right panel were 40-fold. SCO, scopolamine; DONE, donepezil; BV, bee venom; WV, wasp venom.

While SCO treatment caused histological injury in the CA1 region of the hippocampal area, a treatment with WV (250 and 500 μg/kg BW) or BV (50 μg/kg BW) attenuated the SCO-induced hippocampal damage (Figure 9). More specifically, pyramidal cell arrangement in CA1 district was noticeably disrupted in the hippocampus of SCO-treated mice while the treatment with WV or BV significantly improved the abnormality.

### 2.8. WV Activated the Nrf2/HO-1 Axis

**Figure 10:**
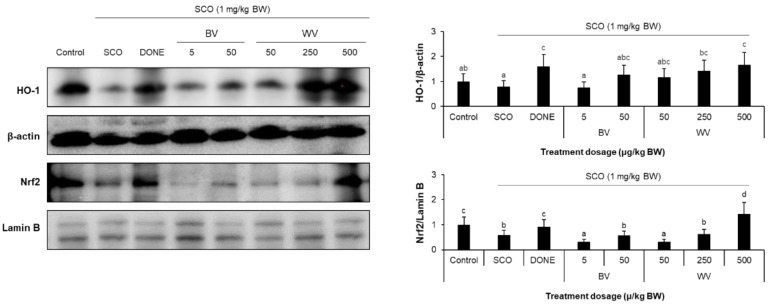
**Effect of WV and BV on Nrf2 and HO-1 expression in the mouse hippocampus**. The expression levels of nuclear Nrf2 and cytoplasmic HO-1 in the hippocampus were measured by western blot analysis and normalized by lamin B and β-actin, respectively. Results are expressed as means ± SD (*n* = 5). Statistical analysis was performed by one-way ANOVA, followed by Duncan’s multiple range test. Values not sharing a common alphabetic character represent a significant difference among experimental groups (*p* < 0.05). SCO, scopolamine; DONE, donepezil; BV, bee venom; WV, wasp venom.

Mice treated with WV at 500 μg/kg BW showed increased nuclear translocation of Nrf2 and subsequent transcriptional activation of its downstream gene, HO-1 (Figure 10). However, BV did not increase the nuclear level of Nrf2 and the expression of the HO-1 gene relative to the SCO control (Figure 10).

### 2.9. WV Decreased Scopolamine-Induced Oxidative Stress Biomarkers

**Figure 11:**
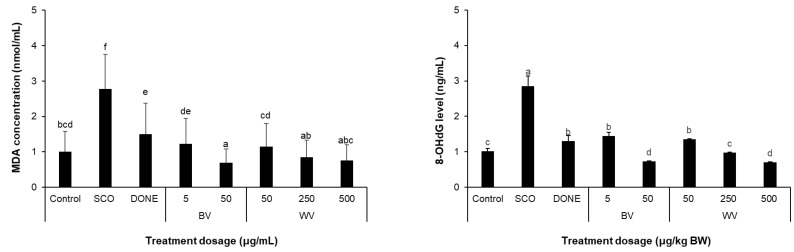
**Effect of BV and WV on the levels of oxidative stress markers.** MDA level in cortex homogenate (**A**) and 8-OHdG level in the plasma (**B**). Results are expressed as means ± SD (*n* = 3 for cortical MDA level; *n* = 6 for plasma 8-OHdG level). Statistical analysis was performed by one-way ANOVA, followed by Duncan’s multiple range test. Values not sharing a common alphabetic character represent a significant difference among experimental groups (*p* < 0.05). SCO, scopolamine; DONE, donepezil; BV, bee venom; WV, wasp venom.

WV treatment significantly reduced the levels of oxidative stress markers in SCO-treated mice (Figure 11). WV administration suppressed the MDA level (lipid peroxidation marker) in cortical homogenates (Figure 11A) and plasma 8-OHdG level (overall DNA damage marker; Figure 11B) in a dose-dependent manner. BV also lowered the levels of those biomarkers of which levels were significantly increased by SCO treatment.

## 3. Discussion

While BV has been widely utilized as an acupuncture agent for the treatment of rheumatoid arthritis and osteoarthritis in Korea, WV is not well-studied for its clinical usefulness [21]. Although hymenoptera venoms can elicit both local and systemic allergic reactions, including life-threatening anaphylaxis, venom immunotherapy remains the most effective treatment reducing the risk of systemic reactions in individuals with hymenoptera venom allergy [22]. In addition, phospholipase A2 which is also found in BV was reported to have the potential to inhibit the progression of AD in the 3 × Tg AD mouse model presumably through the increase in regulatory T cell population [23].

WV has been reported to possess some pharmacological effects in the treatment of pain, inflammatory and neurodegenerative diseases [7,24,25]. Our previous study showed that WV exhibited strong antiinflammatory activity, although the effective dose was higher than the dose given of BV [6]. The present study is a follow-up to our previous work, and aimed to examine if WV could attenuate SCO-induced learning and memory impairment through antioxidant enzyme induction in the mouse model.

Multiple studies have indicated that Nrf2, a master regulator of inducible antioxidant enzymes, is involved in suppressing inflammatory responses and ROS generation in the brain, and thereby its activation can be utilized as a preventive measure from neurodegenerative disorders [8,26]. In particular, hallmarks of AD include an accumulation of senile plaques mainly consisting of fibrillary Aβ peptide, dystrophic neurites, and neurofibrillary tangles composed of hyperphosphorylated tau protein in the brain, leading to dysfunction and loss of synapses and eventual neuronal death [27,28]. Although the etiology of AD remains unknown, oxidative stress is presumed to play a key role in initiation and progression of the disease [29]. In this regard, our study investigated whether WV can promote antioxidant response by activating the Nrf2/HO-1 axis in the hippocampus and thus improve oxidative stress-induced cognitive damage in the SCO-treated mouse model.

Results from the behavioral tasks demonstrated that the administration of WV to mice at the doses of 50 to 500 μg/kg BW ameliorated SCO-induced spatial and associative learning and memory impairments. It has been known that oxidative stress raised in the mammalian brain contributes to cognitive impairment in experimental animals as well as human [13], and that SCO treatment can provoke oxidative stress in the brain and result in learning and memory deficits [14,15]. Thus, SCO-treated mice or rats are often used as a dementia model.

Interestingly enough, WV induced ARE-luciferase reporter activity in a concentration-dependent manner, suggesting that the presence of WV activated the Nrf2 signaling pathway and its downstream antioxidant enzyme genes like *HO-1*. Consistently, the expression of HO-1 protein was upregulated in HT22 cells and hippocampal tissue in mice. According to the literature, ROS production was inversely proportional to the expression level of *HO-1* [30]. HO-1 catalytically converts heme to carbon monoxide and biliverdin, and biliverdin is subsequently metabolized to bilirubin which works as a strong biological antioxidant in mammalian cells. Therefore, we speculate that the protective effect of WV from oxidative stress-induced hippocampal neuronal cell death and memory disruption was mediated by Nrf2-dependent induction of antioxidant enzymes.

The effective dose of WV on antioxidant activity and cognitive function was approximately 10-fold higher than BV as assessed by cultured cell and mouse models. In addition, the cytotoxicity of WV was about 10-fold lower than BV. Therefore, it is presumed that bioactive ingredient(s) in WV is diluted by about 10-fold compared to BV. However, further study is needed to determine appropriate dosage regimens for the clinically beneficial effects in human health.

Although this study supports the memory-improving effect of WV in a mouse model, the active compounds responsible for cognitive enhancement remains unclear. WV reportedly contains a variety of biologically active constituents, including biogenic amines, enzymes, allergens, bioactive peptides, and many others [31]. Similar to BV, it is most likely that small molecule(s) in WV would exert a neuroprotective effect against SCO insult in the mouse brain and hippocampus via activation of the Nrf2 signaling pathway. We attempted to purify the WV component(s) involved in cognitive improvement using bioassay (ARE-luciferase reporter assay in HT22-ARE cells)-guided fractionation and succeeded in identifying serotonin as a potential bioactive component. Serotonin was found to efficiently suppress ROS production induced by *tert*-butyl hydroperoxide (*t*BHP) in mouse hippocampal HT22 cells (Supplementary Figure S1). It is consistent with the findings from a study by Liu and colleagues that WV exhibited antioxidant activity in human keratinocyte against oxidative stress, and that serotonin was identified as the major compound [32].

However, it is generally believed among scientists that serotonin rarely cross the blood-brain barrier (BBB), and therefore, it is uncertain yet whether serotonin found in WV is one of the bioactive compounds primarily responsible for improving the learning and memory function in mice. Based on previous research, serotonin may convert to BBB-permeable metabolite(s) such as 5-hydroxy tryptophan or melatonin in tissue or in blood before being transported to the brain [33,34] and consequently the metabolite(s) might have produced the effect. Another possibility is an indirect effect of serotonin-containing WV which was intraperitoneally administered to mice. Serotonin in mammals is mainly produced by enterochromaffin cells in the gut (about 90% of total serotonin) while the remaining part is synthesized in the brain [35,36]. Peripheral serotonin is actively taken up by platelets and released on their activation in blood and, moreover, contributes to a variety biological functions including immune responses and energy balance through the gut-brain axis [37,38]. Thus, it is conceived that WV-derived serotonin or its metabolites would indirectly influence the memory function in the brain via yet unknown mechanism(s).

## 4. Conclusions

In conclusion, we found that WV restored SCO-induced learning and memory impairment partly through activation of the Nrf2/HO-1 signaling pathway and subsequently increased antioxidant potential. However, further studies on the identification and working mechanism of bioactive component(s) in WV which are responsible for memory-enhancing effect in the SCO-induced amnesic mouse model are needed.

## 5. Materials and Methods

### 5.1. Preparation of WV and BV

*V. velutina* colonies were collected in South Korea during August and October of 2019, and were stored at −80°C until needed. The venom sample was filtered through a Spin-X 0.45-μm cellulose acetate centrifuge tube filter (Corning Inc., Salt Lake City, UT, USA) after manual removing the venom sac from each wasp. The filtrate was then freeze-dried. The details and yield of WV sample preparation are described in our previous report [6]. BV powder was purchased from Chung Jin Biotech Co., Ltd. (Ansan, South Korea). The lyophilized WV and BV were dissolved in dimethyl sulfoxide (DMSO; Dongin Biotech, Seoul, South Korea) at a stock concentration of 100 mg/mL for further examinations.

### 5.2. Cell Culture

The mouse hippocampal neuronal cell line, HT22, was obtained from Prof. Dong-Seok Lee at Kyungpook National University (Daegu, South Korea). HT22 cells (passages 18–26) were grown in Dulbecco’s Modified Eagle Medium (DMEM; Welgene, Gyeongsan, South Korea) supplemented with 10% (*v*/*v*) heat-inactivated fetal bovine serum (FBS; Thermo Fisher Scientific, Waltham, MA, USA) and 1% (*v*/*v*) penicillin-streptomycin (100×; Welgene) in a humidified CO_2_ incubator (MCO-19 AIC, Sanyo, Osaka, Japan) at 37 °C and 5% CO_2_/95% air. The cells were subcultured when the confluency reached about 70% using 0.05% trypsin-ethylenediamine tetraacetic acid (EDTA) sodium salt solution (Welgene).

### 5.3. Free Radical Scavenging Assays

The 2,2’-diphenthyl-1-picryhydrazyl (DPPH) and 2,2-azino-bis(3-ethylbenzoline-6-sulphonic acid) (ABTS^+^) were obtained from Sigma-Aldrich (St, Louis, MO, USA). The free radical scavenging activities of WV and BV were measured as previously described [39,40,41] and ascorbic acid was used as a positive control for both assays. The absorbance (Abs) was measured at 515 nm and 734 nm for DPPH and ABTS+ assays, respectively, using a spectrophotometer (Tecan, Grödig, Austria). The radical scavenging activity was calculated as follows: scavenging capability (%) = (1 − Abs_sample_/Abs_blank_) × 100 where Abs_sample_ indicates the absorbance of ascorbic acid or venom samples.

### 5.4. Cell Viability

HT22 cells were plated at a density of 3 × 10^3^ cells per well in a 96-well culture plate and maintained in 10% (*v*/*v*) FBS-containing DMEM for 24 h. Cells were then treated with WV and BV for 24 h in the absence and presence of 5 mM glutamate. Cell viability was determined by the cell counting kit-8 (CCK-8; Dojindo Laboratories, Kumamoto, Japan) as per the protocol supplied by the manufacturer. The relative cell viability is presented as the percentage of untreated cells.

### 5.5. ARE-Luciferase Reporter Assay

The HT22 cells (passage 8) transduced with pGL4.37[luc2P/ARE/Hyg] vector (Promega Corp., Madison, MA, USA) were stably established and named as HT22-ARE cell line as previously described [42,43,44]. Briefly, HT22 cells were plated at a density of 5 × 10^4^ cells per well of 6-well culture plate and transfected with 100 ng of the vector containing luciferase-encoding gene following the ARE sequence using Lipofectamine^TM^ 2000 Transfection Reagents. The transfectant cells were then selected by clonal growth in the maintenance medium containing 400 μM hygromycin B (Sigma-Aldrich).

For ARE-luciferase reporter assay, HT22-ARE cells (passages between 12–18 after transfection) were plated at a density of 5 × 10^5^ cells per well in a 6-well plate and treated with WV and BV for 24 h. Sulforaphane (1 μM) was used as a positive control. The cells were then harvested and subjected to luciferase assay system (Promega Corp., Madison, WI, USA) as per the manufacturer’s instruction. Briefly, the harvested cells were lysed using the provided lysis buffer. The lysates were mixed with the luciferase assay substrate, luciferin. The reaction mixture was transferred to each well of a 96-well plate. The luminescence was determined using a Glomax 96 microplate luminometer (Promega Corp.). Each value was normalized to its corresponding total protein. The results were averaged and calculated relative to the control.

### 5.6. Determination of Intracellular ROS Level

The cell permeant dye, 2’,7’-dichlorodihydrofluorescein diacetate (H_2_DCFDA; Sigma-Aldrich), can be deacetylated by cellular esterase and later oxidized by ROS to produce dichlorofluorescein (DCF) which is highly fluorescent. The intracellular ROS level can thus be assessed based on the level of fluorescence after treatment with H_2_DCFDA [15,43]. To measure the intracellular ROS level, HT22 cells were plated at a density of 3 × 10^3^ cells per well in a 96-well black polystyrene plate (Nunc, Rochester, NY, USA) or at a density of 3 × 10^4^ cells per well in a 24-well transparent plate containing a 12-mm coverglass pre-coated with 10% poly-L-lysine solution. After treatment with 5 mM glutamate and/or venom sample for 6 h, the cells were loaded with 20 μM H_2_DCFDA at 37 °C for 30 min. The fluorescence of intracellular DCF was quantified using a fluorescence microplate reader (Tecan, Grödig, Austria) at excitation and emission wavelengths of 485 nm and 535 nm, respectively. The fluorescence for each condition was expressed as a fold change relative to the control. The cells placed on the coverglass were mounted onto the microscope slide (Thermo Fisher Scientific), and fluorescent images were then taken by a fluorescence microscope (Eclipse TE2000-U; Nikon, Tokyo, Japan).

### 5.7. Western blotting

HT22 cells were plated at a density of 5 × 10^5^ cells in a 100-mm dish and treated with BV (1 and 2 μg/mL) or WV (10 and 20 μg/mL) for 24 h. The harvested cells were lysed and subjected to fractionation of nuclear and cytoplasmic proteins using NE-PER™ Nuclear and Cytoplasmic Extraction Reagents (Thermo Fisher Scientific). The extracted fractions were quantified by Bradford assay and the equal amount of proteins was loaded and electrophoretically separated onto 10% sodium dodecyl sulfate polyacrylamide gel. The proteins were then transferred to polyvinylidene fluoride (PVDF) membranes (Millipore, Burlingron, MA, USA). Protein bands on the PVDF membranes were allowed to react sequentially with primary antibodies (Abcam, Cambridge, UK) against HO-1 and β-actin in cytoplasmic fraction, and those for Nrf2 and lamin B in nuclear fraction and the appropriate secondary antibodies conjugated with horseradish peroxidase (Thermo Fisher Scientific). The antibody-bound proteins were visualized using the SuperSignal^TM^ West Femto PLUS Chemiluminescent Substrate Kit (Thermo Fisher Scientific) and ImageQuant LAS 4000 mini (GE Healthcare Life Sciences, Little Chalfont, UK). Intensities of protein bands were determined by Image Studio Lite version 5.2 (LI-COR Biotechnology, Lincoln, NE, USA).

For the preparation of protein samples from the animal tissues, the whole brain was dissected from the sacrificed mice and the hippocampal area was removed. The hippocampal tissues were homogenized and subsequently processed for fractionation and Western blotting as mentioned above.

### 5.8. Animal Experiment

The animal study was conducted according to the guidelines of the Institutional Animal Care and Use Committee of Kyungpook National University (approval number: KNU 2021-007). Each group was subjected to histological analysis (*n* = 2) and biochemical assays (*n* = 5) otherwise stated. C57BL/6J mice (6-week old, male) were obtained from Orient Bio Inc. (Seongnam, South Korea). After a week of acclimation, a total of 56 mice were allocated into 8 treatment groups (7 mice per group) as follows: (1) a group received vehicle only; (2) a group received SCO (Sigma-Aldrich) alone at a dose of 1 mg/kg body weight (BW); (3) a group received SCO and Donepezil (Sigma-Aldrich) at 5 mg/kg BW as a positive control; (4) a group received SCO and BV at 5 μg/kg BW; (5) a group received SCO and BV at 50 μg/kg BW; (6) a group received SCO and WV at 50 μg/kg BW; (7) a group received SCO and WV at 250 μg/kg BW; and (8) a group received SCO and WV at 500 μg/kg BW. Lyophilized BV and WV were dissolved in DMSO. Vehicle was normal saline (Sigma-Aldrich) containing 0.5% (*v*/*v*) DMSO and 5% (*v*/*v*) Tween^®^ 80. SCO and venom samples were all intraperitoneally injected every day. BV and WV were injected 15 h prior to the SCO injection on a daily basis for the whole experimental period (refer to Figure 6). Behavioral tests were conducted 30 min after SCO treatment. At termination of scheduled experiments, all mice were sacrificed by asphyxiation in a CO_2_ chamber and dissected for brain and liver tissues and blood.

### 5.9. Behavioral Test

The learning and memory impairment behavior was tested by the Y-maze, passive avoidance, and Morris water maze tasks according to the procedures described in our previous reports [15,42,44,45,46,47,48].

The passive avoidance test was performed in the testing apparatus (Gemini Avoidance System, San Diego, CA, USA) composed of two chambers and a guillotine door. On the first day (Experimental Day 2; refer to Figure 6), every mouse was adapted in the apparatus by placing it in the bright chamber and allowing to move back and forth to the dark chamber for 1 min. On the second day, each mouse was placed in the bright chamber. When the mouse moved to the dark chamber, an electrical food shock (0.5 mA) was delivered for 3 s after the door closed. On the third day (Experimental Day 4), each mouse was again placed in the bright chamber and the latency time for a mouse to stay in the bright chamber was acquired. The latency over 5 min was clocked as 300 s.

The Morris water maze test was performed in a circular water pool (90 cm in diameter and 45 cm in height; colored with nontoxic paint). On the first day (Experimental Day 5), each mouse was allowed to freely swim for 60 s. On the next day, a platform was submerged in one of the pool quadrants. For a consecutive three days (Experimental Day 6–8), mice were given three trials per session per day to search for the platform in place. If the mice did not locate the platform in 60 s, it was guided to place on the platform and allowed to stay for 10 s. On the fifth day (Experimental Day 9), the swimming time until a mouse arrived the platform was recorded.

The Y-maze test was performed in the Y-shaped maze having three arms on Experimental Day 10. Each mouse was placed in the A arm, and its alternations among the arms were monitored and recorded. Spontaneous alternations were defined as consecutive triplets of different arm entries. The percentage of alternations was calculated as follows: Spontaneous alternation (%) = [number of alternations/(total arm entries −2)] × 100.

### 5.10. Histological Analysis by Hematoxylin and Eosin (H&E) Staining

Collected brain tissues were immediately fixed in formalin solution (Sigma-Aldrich) and embedded in paraffin, as previously described [49]. The paraffin blocks were then sectioned at a thickness of 5 μm using a microtome (RM-2025 RT; Leica, Nussloch, Germany). The sections including parts of the hippocampi were placed on Superfrost PLUS microscope slides (Marienfeid, Lauda-Konigshfen, Germany), air-dried at 37 °C for 12 h, and stored at 4 °C before being processed for H&E staining as previously described [15,42,48].

### 5.11. Measurement of Plasma 8-hydroxy-2′-deoxyguanosine (8-OHdG) Level

To determine the level of 8-OHdG in the plasma, a biomarker for oxidative DNA damage, whole blood was collected from the mice into microcentrifuge tube coated with 10 unit of heparin (Sigma) and centrifuged at 2500× *g* for 15 min using a centrifuge (Gyrogen, Gimpo, South Korea). The plasma was subjected to quantification of 8-OHdG using an ELISA kit (Cat# ADI-EKS-350; Enzo Life Sciences International Inc., Plymouth Meeting, PA, USA) according to the manufacturer’s instructions.

### 5.12. Determination of Lipid Peroxidation in Cerebral Cortex Tissues

The brain was dissected from the mice and its cerebral cortex was removed. The cortical tissues were manually homogenized in cold phosphate-buffered saline on ice and subjected to quantification of malondialdehyde (MDA), a biomarker for lipid peroxidation, by a thiobarbituric acid reactive substance (TBARS) assay using an Oxi-TEK TBARS Assay Kit (Cat# ALX-850-287-KI01; Enzo Life Science, Inc., NY, USA) according to the protocol supplied by the manufacturer.

### 5.13. Statistical Analysis

The obtained data were analyzed by one-way analysis of variance (ANOVA) and Duncan’s multiple range test using the Statistical Package for the Social Sciences (SPSS) 25 software (SPSS Inc., Chicago, IL, USA). Comparisons between two groups were performed using Student’s unpaired *t*-test, and *p*-values less than 0.05 were considered significant. Statistical differences were indicated using different alphabetical letters.

## Figures and Tables

**Table 1:** Experimental groups for animal study.

Group No.	Treatment	
	Sample	SCO (1 mg/kg BW)
1	Vehicle	–
2	Vehicle	+
3	Donepezil (5 mg/kg BW)	+
4	BV at 5 μg/kg BW	+
5	BV at 50 μg/kg BW	+
6	WV at 50 μg/kg BW	+
7	WV at 250 μg/kg BW	+
8	WV at 500 μg/kg BW	+

## Data Availability

All data generated or analyzed during this study are included in this published article and its Supplementary Materials.

## Supplementary Materials

The following supporting information can be downloaded at: https://www.mdpi.com/article/10.3390/toxins14040256/s1, Figure S1: Reduction of *t*BHP-induced ROS production by treatment of HT22 cells with WV, BV, or seroton.

## References

[1] Monteiro M.C., Romao P.R., Soares A.M. (2009). Pharmacological perspectives of wasp venom. Protein Pept. Lett..

[2] Kim Y., Son M., Noh E.Y., Kim S., Kim C., Yeo J.H., Park C., Lee K.W., Bang W.Y. (2016). MP-V1 from the Venom of Social Wasp Vespula vulgaris Is a de Novo Type of Mastoparan that Displays Superior Antimicrobial Activities. Molecules.

[3] Chen W.H., Yang X.B., Yang X.L., Zhai L., Lu Z.K., Liu J.Z., Yu H.N. (2008). Antimicrobial peptides from the venoms of Vespa bicolor Fabricius. Peptides.

[4] Wu R., Li D., Tang Q., Wang W., Xie G., Dou P. (2018). A Novel Peptide from Vespa ducalis Induces Apoptosis in Osteosarcoma Cells by Activating the p38 MAPK and JNK Signaling Pathways. Biol. Pharm. Bull..

[5] Danneels E.L., Gerlo S., Heyninck K., Van Craenenbroeck K., De Bosscher K., Haegeman G., de Graaf D.C. (2014). How the venom from the ectoparasitoid Wasp nasonia vitripennis exhibits anti-inflammatory properties on mammalian cell lines. PLoS ONE.

[6] Yun H.S., Oh J., Lim J.S., Kim H.J., Kim J.S. (2021). Anti-Inflammatory Effect of Wasp Venom in BV-2 Microglial Cells in Comparison with Bee Venom. Insects.

[7] Silva J., Monge-Fuentes V., Gomes F., Lopes K., dos Anjos L., Campos G., Arenas C., Biolchi A., Goncalves J., Galante P. (2015). Pharmacological Alternatives for the Treatment of Neurodegenerative Disorders: Wasp and Bee Venoms and Their Components as New Neuroactive Tools. Toxins.

[8] Chen Z., Zhong C. (2014). Oxidative stress in Alzheimer’s disease. Neurosci. Bull..

[9] Klein J.A., Ackerman S.L. (2003). Oxidative stress, cell cycle, and neurodegeneration. J. Clin. Investig..

[10] Sayre L.M., Perry G., Smith M.A. (2008). Oxidative stress and neurotoxicity. Chem. Res. Toxicol..

[11] Bhat S.A., Sood A., Shukla R., Hanif K. (2019). AT2R Activation Prevents Microglia Pro-inflammatory Activation in a NOX-Dependent Manner: Inhibition of PKC Activation and p47(phox) Phosphorylation by PP2A. Mol. Neurobiol..

[12] Muche A., Arendt T., Schliebs R. (2017). Oxidative stress affects processing of amyloid precursor protein in vascular endothelial cells. PLoS ONE.

[13] Liu R., Liu I.Y., Bi X., Thompson R.F., Doctrow S.R., Malfroy B., Baudry M. (2003). Reversal of age-related learning deficits and brain oxidative stress in mice with superoxide dismutase/catalase mimetics. Proc. Natl. Acad. Sci. USA.

[14] Fan Y., Hu J., Li J., Yang Z., Xin X., Wang J., Ding J., Geng M. (2005). Effect of acidic oligosaccharide sugar chain on scopolamine-induced memory impairment in rats and its related mechanisms. Neurosci. Lett..

[15] Ju S., Seo J.Y., Lee S.K., Oh J., Kim J.S. (2021). Oral administration of hydrolyzed red ginseng extract improves learning and memory capability of scopolamine-treated C57BL/6J mice via upregulation of Nrf2-mediated antioxidant mechanism. J. Ginseng Res..

[16] Murphy T.H., Miyamoto M., Sastre A., Schnaar R.L., Coyle J.T. (1989). Glutamate toxicity in a neuronal cell line involves inhibition of cystine transport leading to oxidative stress. Neuron.

[17] Stanciu M., Wang Y., Kentor R., Burke N., Watkins S., Kress G., Reynolds I., Klann E., Angiolieri M.R., Johnson J.W. (2000). Persistent activation of ERK contributes to glutamate-induced oxidative toxicity in a neuronal cell line and primary cortical neuron cultures. J. Biol. Chem..

[18] Kritis A.A., Stamoula E.G., Paniskaki K.A., Vavilis T.D. (2015). Researching glutamate-induced cytotoxicity in different cell lines: A comparative/collective analysis/study. Front. Cell. Neurosci..

[19] Tobaben S., Grohm J., Seiler A., Conrad M., Plesnila N., Culmsee C. (2011). Bid-mediated mitochondrial damage is a key mechanism in glutamate-induced oxidative stress and AIF-dependent cell death in immortalized HT-22 hippocampal neurons. Cell Death Differ..

[20] Lalonde R. (2002). The neurobiological basis of spontaneous alternation. Neurosci. Biobehav. Rev..

[21] Lee J.D., Park H.J., Chae Y., Lim S. (2005). An Overview of Bee Venom Acupuncture in the Treatment of Arthritis. Evid.-Based Complement. Altern. Med..

[22] Ozdemir C., Kucuksezer U.C., Akdis M., Akdis C.A. (2011). Mechanisms of immunotherapy to wasp and bee venom. Clin. Exp. Allergy.

[23] Ye M., Chung H.S., Lee C., Yoon M.S., Yu A.R., Kim J.S., Hwang D.S., Shim I., Bae H. (2016). Neuroprotective effects of bee venom phospholipase A2 in the 3xTg AD mouse model of Alzheimer’s disease. J. Neuroinflamm..

[24] Gao Y., Yu W.X., Duan X.M., Ni L.L., Liu H., Zhao H.R., Xiao H., Zhang C.G., Yang Z.B. (2020). Wasp Venom Possesses Potential Therapeutic Effect in Experimental Models of Rheumatoid Arthritis. Evid.-Based Complement. Altern. Med..

[25] Dongol Y., Dhananjaya B.L., Shrestha R.K., Aryal G. (2016). Wasp Venom Toxins as a Potential Therapeutic Agent. Protein Pept. Lett..

[26] Bahn G., Jo D.G. (2019). Therapeutic Approaches to Alzheimer’s Disease Through Modulation of NRF2. Neuromol. Med..

[27] Nelson P.T., Braak H., Markesbery W.R. (2009). Neuropathology and cognitive impairment in Alzheimer disease: A complex but coherent relationship. J. Neuropathol. Exp. Neurol..

[28] Martins R.N., Villemagnen V., Sohrabi H.R., Chatterjee P., Shah T.M., Verdile G., Fraser P., Taddei K., Gupta V.B., Rainey-Smith S.R. (2018). Alzheimer’s Disease: A Journey (f)rom Amyloid Peptides and Oxidative Stress, to Biomarker Technologies and Disease Prevention Strategies-Gains from AIBL and DIAN Cohort Studies. J. Alzheimers Dis..

[29] Butterfield D.A., Halliwell B. (2019). Oxidative stress, dysfunctional glucose metabolism and Alzheimer disease. Nat. Rev. Neurosci..

[30] Vasavda C., Kothari R., Malla A.P., Tokhunts R., Lin A., Ji M., Ricco C., Xu R., Saavedra H.G., Sbodio J.I. (2019). Bilirubin Links Heme Metabolism to Neuroprotection by Scavenging Superoxide. Cell Chem. Biol..

[31] Abd El-Wahed A., Yosri N., Sakr H.H., Du M., Algethami A.F.M., Zhao C., Abdelazeem A.H., Tahir H.E., Masry S.H.D., Abdel-Daim M.M. (2021). Wasp Venom Biochemical Components and Their Potential in Biological Applications and Nanotechnological Interventions. Toxins.

[32] Le T.N., Da Silva D., Colas C., Darrouzet E., Baril P., Leseurre L., Maunit B. (2020). Asian hornet Vespa velutina nigrithorax venom: Evaluation and identification of the bioactive compound responsible for human keratinocyte protection against oxidative stress. Toxicon Off. J. Int. Soc. Toxinol..

[33] Jones L.A., Sun E.W., Martin A.M., Keating D.J. (2020). The ever-changing roles of serotonin. Int. J. Biochem. Cell B.

[34] Lee B.H., Hille B., Koh D.S. (2021). Serotonin modulates melatonin synthesis as an autocrine neurotransmitter in the pineal gland. Proc. Natl. Acad. Sci. USA.

[35] Jenkins T.A., Nguyen J.C., Polglaze K.E., Bertrand P.P. (2016). Influence of Tryptophan and Serotonin on Mood and Cognition with a Possible Role of the Gut-Brain Axis. Nutrients.

[36] Mosienko V., Bert B., Beis D., Matthes S., Fink H., Bader M., Alenina N. (2012). Exaggerated aggression and decreased anxiety in mice deficient in brain serotonin. Transl. Psychiatry.

[37] Szoke H., Kovacs Z., Bokkon I., Vagedes J., Szabo A.E., Hegyi G., Sterner M.G., Kiss A., Kapocs G. (2020). Gut dysbiosis and serotonin: Intestinal 5-HT as a ubiquitous membrane permeability regulator in host tissues, organs, and the brain. Rev. Neurosci..

[38] El-Merahbi R., Loffler M., Mayer A., Sumara G. (2015). The roles of peripheral serotonin in metabolic homeostasis. FEBS Lett..

[39] Re R., Pellegrini N., Proteggente A., Pannala A., Yang M., Rice-Evans C. (1999). Antioxidant activity applying an improved ABTS radical cation decolorization assay. Free Radic. Biol. Med..

[40] Averilla J.N., Oh J., Wu Z., Liu K.H., Jang C.H., Kim H.J., Kim J.S., Kim J.S. (2019). Improved extraction of resveratrol and antioxidants from grape peel using heat and enzymatic treatments. J. Sci. Food Agric..

[41] Woo Y., Lee H., Jeong Y.S., Shin G.Y., Oh J.G., Kim J.S., Oh J. (2017). Antioxidant Potential of Selected Korean Edible Plant Extracts. BioMed Res. Int..

[42] Seo J.Y., Ju S.H., Oh J., Lee S.K., Kim J.S. (2016). Neuroprotective and Cognition-Enhancing Effects of Compound K Isolated from Red Ginseng. J. Agric. Food Chem..

[43] Kim M.S., Seo J.Y., Oh J., Jang Y.K., Lee C.H., Kim J.S. (2017). Neuroprotective Effect of Halophyte Salicornia herbacea L. Is Mediated by Activation of Heme Oxygenase-1 in Mouse Hippocampal HT22 Cells. J. Med. Food.

[44] Seo J.Y., Kim B.R., Oh J., Kim J.S. (2018). Soybean-Derived Phytoalexins Improve Cognitive Function through Activation of Nrf2/HO-1 Signaling Pathway. Int. J. Mol. Sci..

[45] Seo J.Y., Lim S.S., Kim J., Lee K.W., Kim J.S. (2017). Alantolactone and Isoalantolactone Prevent Amyloid beta25-35 -induced Toxicity in Mouse Cortical Neurons and Scopolamine-induced Cognitive Impairment in Mice. Phytother. Res. PTR.

[46] Kim S., Oh J., Jang C.H., Kim J.S. (2019). Improvement of cognitive function by Gochujang supplemented with tomato paste in a mouse model. Food Sci. Biotechnol..

[47] Woo Y., Lim J.S., Oh J., Lee J.S., Kim J.S. (2020). Neuroprotective Effects of Euonymus alatus Extract on Scopolamine-Induced Memory Deficits in Mice. Antioxidants.

[48] Lee S., Lim J.S., Yun H.S., Kim Y., Jeong S., Hwang S.D., Kim J.W., Oh J., Kim J.S. (2021). Dietary supplementation with Ceriporia lacerata improves learning and memory in a scopolamine-induced amnesia mouse model. Food Sci. Biotechnol..

[49] Saintemarie G. (1962). A Paraffin Embedding Technique for Studies Employing Immunofluorescence. J. Histochem. Cytochem..

