# Supplementary material for: Wasp Venom Ameliorates Scopolamine-Induced Learning and Memory Impairment in Mice
Source: Toxins (Basel). 2022 Apr 4;14(4):256. doi: 10.3390/toxins14040256 (PMC9029392; doi:10.3390/toxins14040256)
Supplement: Supplementary file 1 [file toxins-14-00256-s001.zip › toxins-1637386-supplementary.pdf]

Wasp Venom Ameliorates Scopolamine-Induced Learning and Memory Impairment in Mice

Ji Hyeong Chae, Jisun Oh, Ji Sun Lim, Yoon Ah Jeong, Hyun Seok Yun, Chan Ho Jang, Hyo Jung Kim and Jong-Sang Kim

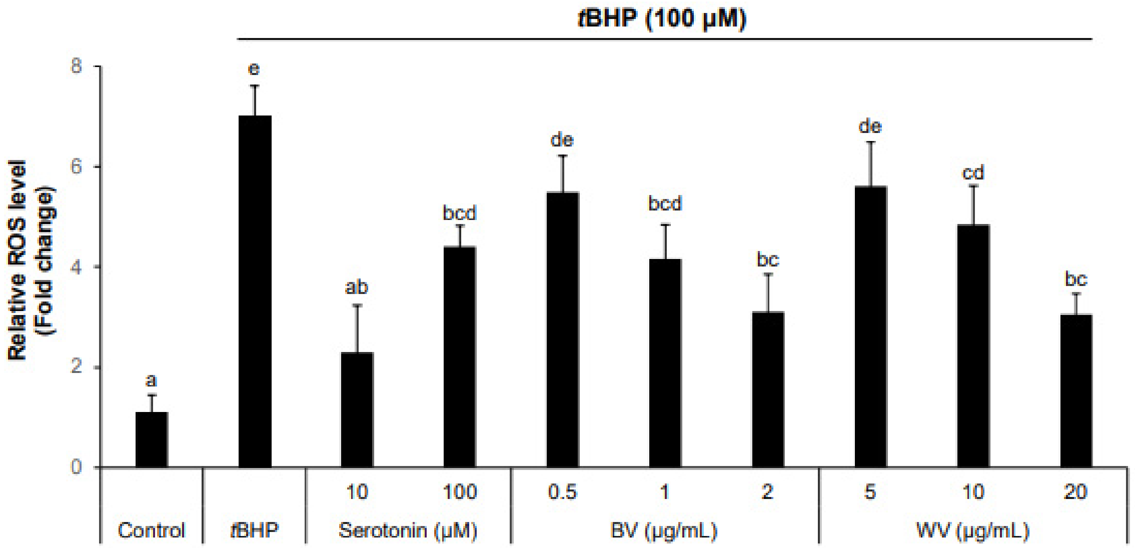

Figure S1. Reduction of *t*BHP-induced ROS production by treatment of HT22 cells with WV, BV, or serotonin. HT22 cells were treated with BV, WV, or serotonin in the presence of *t*BHP. Intracellular ROS level was measured by DCF assay. Results are expressed as means ± SEM ($n = 3$). Statistical analysis was performed by one-way ANOVA, followed by Duncan's multiple range test. Values not sharing a common alphabetic character represent a significant difference among experimental groups ($p < 0.05$).
